# P50 implies adverse clinical outcomes in pediatric acute respiratory distress syndrome by reflecting extrapulmonary organ dysfunction

**DOI:** 10.1038/s41598-022-18038-6

**Published:** 2022-08-11

**Authors:** Yura Kim, Jae Hwa Jung, Ga Eun Kim, Mireu Park, Myeongjee Lee, Soo Yeon Kim, Min Jung Kim, Yoon Hee Kim, Kyung Won Kim, Myung Hyun Sohn

**Affiliations:** 1 Department of Pediatrics, Severance Children’s Hospital, Institute of Allergy, Brain Korea 21 PLUS Project for Medical Science, Yonsei University College of Medicine, 50-1, Yonsei-ro, Seodaemun-gu, Seoul, 03722 Republic of Korea; 2 Department of Pediatrics, Kyung Hee University College of Medicine, Seoul, Republic of Korea; 3 Department of Pediatrics, Keimyung University School of Medicine, Keimyung University Dongsan Hospital, Daegu, Republic of Korea; 4 Biostatistics Collaboration Unit, Department of Biomedical Systems Informatics, Yonsei University College of Medicine, Seoul, Republic of Korea

**Keywords:** Respiratory distress syndrome, Paediatric research

## Abstract

Hypoxemia and multiple organ dysfunction are significant contributors to mortality in patients with pediatric acute respiratory distress syndrome (PARDS). P50, the oxygen tension at which hemoglobin is 50% saturated, is a measure of hemoglobin-oxygen affinity, and its alteration might have implications for tissue hypoxia and organ dysfunction. The purpose of this single-center, retrospective study was to evaluate P50 levels in PARDS and to determine the association between P50 and clinical outcomes. The study included 212 children diagnosed with PARDS according to the Pediatric Acute Lung Injury Consensus Conference definition who required invasive mechanical ventilation and had arterial blood gas results of hemoglobin oxygen saturation < 97% at the time of diagnosis. P50 levels were calculated using Doyle’s method, and organ dysfunction was assessed using the Pediatric Logistic Organ Dysfunction-2 score. Most patients exhibited more than one dysfunctional extrapulmonary organ at PARDS onset. P50 increased with increasing PARDS severity (mild (26.6 [24.9–29.6]), moderate (26.8 [25.0–29.5]), and severe PARDS (29.1 [26.1–32.4] mmHg; *P* = 0.025). Moreover, P50 demonstrated a significant positive association with extrapulmonary organ dysfunction score (β = 0.158, *P* = 0.007) and risk of mortality (adjusted hazard ratio, 1.056; 95% confidence interval, 1.015–1.098; *P* = 0.007), irrespective of initial PARDS severity. The relationship between P50 and mortality was largely mediated by extrapulmonary organ dysfunction. A high P50 value at the time of PARDS diagnosis may be associated with mortality via dysfunctional extrapulmonary organs. Future studies should consider P50 as a potential candidate index for risk stratification of PARDS patients.

## Introduction

Acute respiratory distress syndrome (ARDS) refers to hypoxic respiratory failure due to diffuse pulmonary inflammation. ARDS results in increased capillary endothelial permeability following a variety of direct and indirect injuries to the lung^1^. Although improvements in pediatric critical care support have led to a decrease in mortality rates, pediatric ARDS (PARDS) remains a significant cause of childhood morbidity and mortality worldwide^2–5^.

Of note, the predominant cause of death in PARDS does not appear to be severe hypoxemia, which is one of the defining criteria in PARDS, but multiple organ dysfunction^6,7^. PARDS is a severe lung disease exhibiting complex pathophysiology that involves not only the respiratory system but also the extrapulmonary end-organs. Therefore, extrapulmonary organ dysfunction is common in both adults and children^8–10^. It generally occurs concurrently with respiratory dysfunction; however, additional organ dysfunction may develop after the onset of respiratory failure^11^. Previous studies have consistently demonstrated that extrapulmonary organ dysfunction is a strong risk factor for mortality that is independent of hypoxemia degree, or other respiratory criteria^7,10,12^. The Pediatric Acute Lung Injury Consensus Conference (PALICC) sub-committee has stated that multiple organ dysfunction is ‘the single most important independent clinical risk factor for mortality at the onset of ARDS’^13^. However, the pathophysiological mechanisms linking multiple organ dysfunction to PARDS remain unclear.

At the tissue level, oxygen supply and demand are dependent on various factors including peripheral oxygen delivery, diffusion from microcirculation to tissue mitochondria, and local tissue oxygen consumption. Abnormalities of systemic gas exchange, such as imbalances between oxygen delivery and uptake, have been suggested as potential mechanisms underlying extrapulmonary organ dysfunction in ARDS with a low level of oxygen uptake below the critical threshold^14^. Nearly 98% of circulating oxygen is bound to hemoglobin, and the affinity of hemoglobin for oxygen in relation to the partial pressure of oxygen (pO_2_) is demonstrated by the hemoglobin-oxygen dissociation curve. The oxygen tension at which hemoglobin is 50% saturated (P50) is a measure of the affinity of hemoglobin for oxygen that determines the release of oxygen from microcirculation into tissues^15^. Therefore, alterations in P50 might have implications for tissue hypoxia and subsequent organ dysfunction via altered oxygen extraction^15,16^.

Few studies have investigated P50 in ARDS. In addition, no investigation has reported P50 in relation to multiple organ dysfunction in PARDS. Thus, we aimed to evaluate the level of P50 in PARDS patients, investigate the associations between P50 and various organ dysfunctions, and assess the possible link between P50, extrapulmonary organ dysfunction, and clinical outcomes in patients with PARDS.

## Materials and methods

### Patient selection and P50 calculation

First, we extracted data from the Severance Hospital electronic database and retrospectively reviewed the records of patients aged 1 month to 19 years who were admitted to the intensive care unit (ICU) from January 1, 2010 to January 30, 2017. A total of 1,101 patients were then screened for inclusion in this study. Next, we identified patients with respiratory difficulty requiring invasive mechanical ventilation. Finally, we reviewed medical records to identify if the patient met all the diagnostic criteria of PALICC definition consistent with the timing of onset, etiology for ARDS, chest radiographic images for the presence of pulmonary infiltrates, the origin of edema not fully explained by cardiac failure or fluid overload, and oxygenation parameters. The eligibility criteria were as follows: (1) diagnosis of PARDS according to the PALICC criteria^17^, which requires invasive mechanical ventilation; and (2) at least one documented arterial blood gas result at the time of PARDS diagnosis. Patients with congenital cyanotic heart disease were excluded because these patients were cared for in a separate cardiothoracic intensive care unit.

In vivo P50 values were calculated using the method described by Doyle^18^ based on Hill’s equation^19^ $\left( {{\text{P5}}0 = {\text{pO}}_{2} \times \left( {\frac{{1 - {\text{SO}}_{2} }}{{{\text{SO}}_{2} }}} \right)^{{\frac{1}{{\text{n}}}}} ,\,{\text{n}} = {2}.{711}} \right)$, which uses pO_2_ and hemoglobin oxygen saturation (sO_2_) from a single-point measurement of arterial gases. All arterial gas results were the nearest approximation to the time of PARDS diagnosis and within a maximum of 4 h after diagnosis. Only results with measured sO_2_ < 97% were utilized because sO_2_ values ≥ 97% might lead to erroneous calculations at the extremes of the hemoglobin-oxygenation equilibrium curve^20^. In addition, patients younger than 1 year were excluded from the analysis because fetal to adult hemoglobin switching might not yet fully occurred in this population, and fetal hemoglobin has a higher affinity for oxygen than adult hemoglobin^21^.

This study was approved, and a waiver was obtained from the Institutional Review Board of Yonsei University Health System, Severance Hospital for informed consent (IRB No. 4-2013-0207). The study was carried out according to the Declaration of Helsinki.

### Variable definitions

During the ICU stay, demographics and clinical data were assessed, including PARDS etiology, comorbidity, clinical progress, and outcomes. The Pediatric Index of Mortality 3 scores at ICU admission, and Pediatric Logistic Organ Dysfunction (PELOD)-2 score at the time of PARDS diagnosis were collected. ICU mortality and degree of multiple organ dysfunction were selected as the main outcomes. The PELOD-2 score is generally adopted as a clinically meaningful descriptor for the number and severity of organ dysfunctions in critically ill children^22^. The total score was subdivided into five types of organ dysfunction (neurological, cardiovascular, renal, respiratory, and hematologic) by relevant variables. Similarly, extrapulmonary organ dysfunction was assessed using the non-respiratory PELOD-2^23^, which was calculated by simply removing the corresponding respiratory variables.

Clinical outcomes of PARDS, such as ICU mortality, duration of invasive mechanical ventilation, and ventilator-free days at 28 days post-diagnosis were also collected. Ventilator-free days were determined for survivors by subtracting the total duration of invasive ventilation from 28 days. In patients who required ≥ 28 ventilator days or ICU non-survivors, the number of ventilator-free days was 0.

### Statistical analysis

Categorical data were presented as counts and percentages. Continuous data were tested for normality using the Kolmogorov–Smirnov test and reported as the median with interquartile range. Categorical variables were analyzed using the Chi-square test or Fisher’s exact test, and continuous variables were compared using the Kruskal–Wallis H test with Dunn’s method for multiple comparisons. Correlation between the P50 level and PARDS severity was analyzed with the Spearman’s rank correlation test. Multivariate linear regression analyses controlling for age, sex, weight, comorbidity, and initial PALICC grade were performed to assess the associations between P50 and degree of various organ dysfunctions. Cox’s regression model was employed for survival analysis by adjusting for the aforementioned variables. To investigate the possible mechanism underlying P50 and mortality in PARDS patients, mediation analysis was performed for extrapulmonary organ dysfunction as a potential mediator using the method proposed by Lange et al.^24^. All statistical analyses were conducted using software SPSS Statistics (version 25.0; IBM Corp., Armonk, NY, USA) and R (version 3.5.2; R Foundation for Statistical Computing, Vienna, Austria). Two-tailed *P* value < 0.05 was considered to indicate statistical significance.

### Ethics approval and consent to participate

This study was approved by the Severance Hospital’s Institutional Review Board, and the requirement for informed consent was waived (IRB No. 4-2013-0207).

## Results

### Characteristics of the study population

Of the 288 children with PARDS during the study period, 257 exhibited an arterial blood gas of sO_2_ < 97%. Among them, 45 patients were aged younger than 1 year. The remaining 212 patients were included in the final analysis (Supplementary Fig. 1). The demographics and clinical data of the studied population were stratified according to PARDS severity (Table 1). The subgroups did not differ in age, sex, and weight. The Pediatric Index of Mortality 3 and PELOD-2 scores showed significant differences between groups stratified according to PARDS severity. The scores increased with increasing disease severity. The mortality rate increased with PARDS severity, from mild (26.2%) to moderate (38.9%) to severe (68.3%). Among survivors, there were no significant differences in the ICU length of stay, duration of invasive mechanical ventilation, and ventilator-free days between the severity groups (Table 1).

**Table 1:** Demographics of the study population (N = 212).

	Mild PARDS (n = 80)	Moderate PARDS (n = 72)	Severe PARDS (n = 60)	*p* value
Sex, M (%)	54 (67.5%)	48 (66.7%)	32 (53.3%)	0.172
Age (years)	6.2 [2.0;11.4]	4.8 [2.1;10.9]	6.1 [2.2;12.4]	0.737
Weight (kg)	17.2 [10.5;31.5]	17.2 [13.0;33.0]	18.5 [13.0;45.3]	0.175
PIM 3	5.3 [4.2;18.3]	7.2 [4.7;20.1]	24.9 [9.3;40.9]	< 0.001
PELOD-2	6.0 [4.0;8.0]	6.5 [5.0;9.0]	8.0 [6.0;13.0]	< 0.001
Nonrespiratory PELOD-2	3.0 [1.0;5.0]	3.0 [1.0;6.0]	4.0 [2.0;9.0]	0.009
Presence of extrapulmonary organ dysfunction	74 (92.5%)	64 (88.9%)	57 (95.0%)	0.427
Number of extrapulmonary organ dysfunction	1.5 [1.0;2.0]	2.0 [1.0;2.0]	2.0 [1.0;3.0]	0.033
**Comorbidity, n (%)**				0.003
Airway/Pulmonology	25 (31.3%)	24 (33.3%)	22 (36.7%)	
Oncology	15 (18.8%)	25 (34.7%)	21 (35.0%)	
Neurology	26 (32.5%)	12 (16.7%)	7 (11.7%)	
Genetic syndrome	8 (10.0%)	4 (5.6%)	0 (0%)	
Hepatic failure/Liver transplant	4 (5.0%)	0 (0%)	3 (5.0%)	
None	2 (2.5%)	7 (9.7%)	7 (11.7%)	
**PARDS etiology, n (%)**				0.063
Infectious pneumonia	56 (70.0%)	44 (61.1%)	32 (53.3%)	
Aspiration pneumonia	9 (11.3%)	7 (9.7%)	6 (10.0%)	
Sepsis	14 (17.5%)	19 (26.4%)	15 (25.0%)	
Mortality, n (%)	21 (26.2%)	28 (38.9%)	41 (68.3%)	< 0.001
**In survivals (n = 122)**				
ICU length of stay (days)	11.0 [8.0;18.0]	9.5 [7.0;19.5]	14.0 [6.0;22.5]	0.751
IMV duration (days)	9.0 [7.0;15.5]	8.0 [5.0;17.0]	14.0 [7.5;22.0]	0.366
VFD at day 28 (days)	19.0 [12.5;21.0]	20.0 [11.0;23.0]	14.0 [6.0;20.5]	0.335

Data are given as number (%) or median [interquartile range].

*PARDS* Pediatric acute respiratory distress syndrome, *PIM 3* Pediatric index of mortality 3, *PELOD-2* Pediatric logistic organ dysfunction-2 score, *ICU* Intensive care unit, *IMV* Invasive mechanical ventilation, *VFD* Ventilator-free days.

### P50 levels in children with PARDS

**Figure 1:**
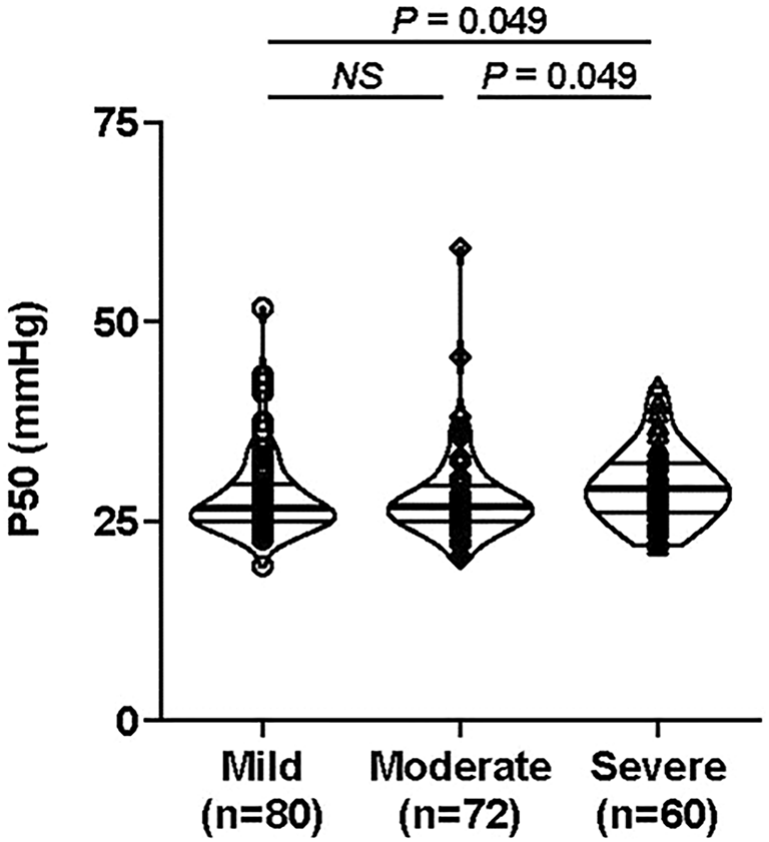
P50 in PARDS patients. Violin plots represent the distribution of P50 levels in mild, moderate, and severe PARDS. Horizontal lines indicate median and interquartile ranges. PARDS; Pediatric acute respiratory distress syndrome.

A trend of increasing P50 levels with increasing PARDS severity was observed (Fig. 1). P50 was significantly higher (*P* = 0.049) in patients with severe PARDS (29.1 [26.1–32.4] mmHg) than in those with mild (26.6 [24.9–29.6] mmHg) or moderate (26.8 [25.0–29.5] mmHg) PARDS. The correlation analysis showed a weak but significant positive correlation between the P50 level and PARDS severity (*r* = 0.153, *P* = 0.026).

### Organ dysfunction in children with PARDS

Most patients (92.0%) exhibited at least one extrapulmonary organ dysfunction at PARDS onset. The number of extrapulmonary organs with dysfunction increased with increasing hypoxemia severity (Table 1). Furthermore, mortality increased with increasing numbers of dysfunctional extrapulmonary organs (Supplementary Fig. 2). Multivariate Cox’s regression analyses demonstrated that PARDS mortality risk was significantly increased with each organ dysfunction included in the nonrespiratory PELOD-2 score, even after adjustment for initial PALICC grade (Supplementary Table 1).

### Association between P50 and organ dysfunction

Multiple regression analyses were used to estimate the regression coefficients of P50 and organ dysfunction scores (Table 2). P50 exhibited significant positive associations with the pulmonary (β = 0.024, *P* = 0.007), extrapulmonary (β = 0.158, *P* = 0.004), and overall organ dysfunction (β = 0.182, *P* = 0.001) scores. Extrapulmonary organ dysfunction was further divided into neurological, cardiovascular, renal, and hematologic dysfunction, which revealed significant associations between cardiovascular and renal dysfunction and P50 (β = 0.075, *P* = 0.001, and β = 0.022, *P* = 0.046, respectively) (Table 2).

**Table 2:** Multivariate regression analysis of the association between P50 and the type of organ dysfunction.

Type of organ dysfunction	Linear regression		
	β coefficient	95% CI	*p* value
Overall	0.182	0.077–0.287	0.001
Pulmonary	0.024	0.007–0.041	0.007
Non-pulmonary	0.158	0.052–0.264	0.004
Neurological	0.066	− 0.002–0.134	0.056
Cardiovascular	0.075	0.029–0.121	0.001
Renal	0.022	0.000–0.043	0.046
Hematologic	− 0.005	− 0.036–0.026	0.742

Analyses were adjusted for age, sex, weight, comorbidities, and initial PALICC grade.

β coefficients were depicted for each mmHg increment of P50.

*CI* Confidence interval, *PALICC* Pediatric acute lung injury consensus conference.

### Association between P50 and clinical outcomes in PARDS

Univariate analysis using Cox’s regression revealed that the mortality risk was significantly increased with increasing P50 levels (hazard ratio [HR] for 1 mmHg change in P50, 1.035; 95% confidence interval [CI], 1.003–1.068; *P* = 0.029). The same association was found after adjustment for potential covariates such as age, sex, weight, comorbidities, and initial PALICC grade (HR, 1.056; 95% CI, 1.015–1.098; *P* = 0.007). In contrast, no significant associations were observed between P50 and ICU length of stay, invasive mechanical ventilation duration, and ventilator-free days (data not shown).

### Putative implications linking P50 to mortality in PARDS

**Figure 2:**
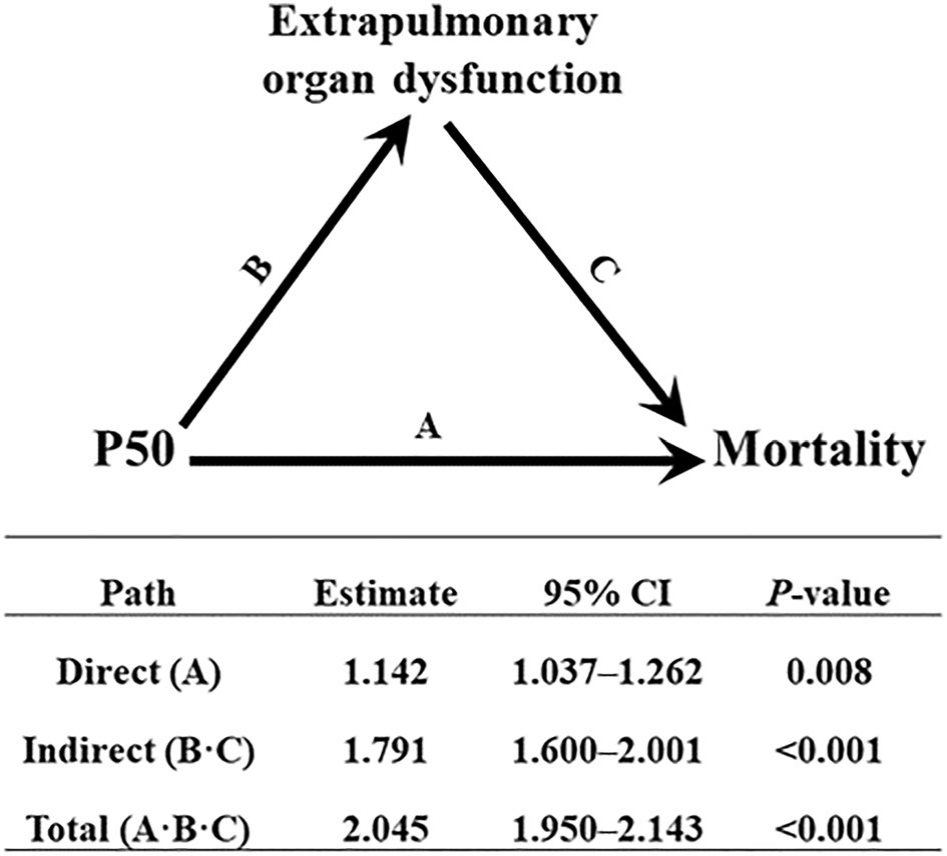
Analysis of the effect of P50 on mortality mediated by extrapulmonary organ dysfunction. The effect estimates on hazard ratio scale and 95% confidence intervals (CI) are tabulated for all paths: direct effect, A; indirect effect, B·C; and total effect, A·B·C. The model is adjusted for age, sex, weight, comorbidity, and initial PALICC grade. Proportion mediation is 81.5% (67.3–94.9%). PALICC; Pediatric Acute Lung Injury Consensus Conference.

The mediation analysis produced models demonstrating that extrapulmonary organ dysfunction partially mediated the relationship between P50 and mortality in PARDS (Fig. 2). After controlling for the aforementioned potential confounders, including initial PALICC grade, P50 was associated with mortality (HR, 2.045; 95% CI, 1.950–2.143; *P* < 0.001). The analysis confirmed a significant direct effect (HR, 1.142; 95% CI, 1.037–1.262; *P* = 0.008) and indirect effect (HR, 1.791; 95% CI, 1.600–2.001; *P* < 0.001) of extrapulmonary organ dysfunction consistent with the presence of mediation. Extrapulmonary organ dysfunction was estimated to mediate 81.5% of the effect of P50 in PARDS mortality.

## Discussion

In this study, P50 levels increased with increasing PARDS severity, with a notable difference in children with severe PARDS. P50 exhibited a significant correlation with the degree of extrapulmonary organ dysfunction as well as respiratory dysfunction. The mortality risk was significantly associated with the P50 level, even after adjusting the initial PARDS severity criteria. Moreover, we demonstrated that extrapulmonary organ dysfunction partially mediated the relationship between P50 and mortality in PARDS patients.

In PARDS patients, extrapulmonary organ dysfunction occurred frequently, with an incidence above 90% at the time of PARDS onset. These findings are consistent with previous studies in children with acute respiratory failure or ARDS. Weiss et al.^11^ reported a prevalence of 63% of concurrent multiple organ dysfunction within a day of the onset of acute respiratory failure, using a multi-centered, prospectively collected dataset of 2449 children. In addition, Kallet et al.^8^ conducted an analysis on 15 years of data from 1747 adults with ARDS and demonstrated that at least one dysfunctional extrapulmonary organ system was present in 80–90% of patients. This pervasive early-onset extrapulmonary organ dysfunction further supports the concept of PARDS as a pulmonary manifestation of a systemic syndrome^9,25^.

The exact nature of the association between lung injury and extrapulmonary organ failure in ARDS remains largely unknown. ARDS could be both the cause and the consequence of multiple organ dysfunction^9^. In ARDS, the redistribution of cardiac output or a loss of recruitable capillary reserve may compromise perfusion within individual organs. Direct endothelial injury and the ensuing alveolar edema increase the diffusion distance for oxygen from the alveoli to the capillaries, and parenchymal damage may impair oxygen utilization^14^. Furthermore, the concept of systemic microcirculatory injury has been supported by the presence of globally activated intravascular inflammatory mediators in ARDS^26–28^. Other than the lung, a primary target organ, the inflammatory process may spill over into the systemic circulation, resulting in widespread endothelial and parenchymal damage. This may cause altered gas exchange in distal organs in an analogous manner as pulmonary injury and dysfunction^29,30^.

Our results have demonstrated that the degree of extrapulmonary organ dysfunction and the number of dysfunctional organ systems increased as the PARDS severity increased. All extrapulmonary organ dysfunctions were closely related to the mortality risk, and the greater the number of dysfunctional organs, the higher the mortality rate. Indeed, mortality in critically ill children results from the interactions among multiple failing organs^31,32^. The pathology of multiple organ dysfunction includes the primary insult, which evokes the host response of the systemic inflammatory process, and the resultant phenomenon of 'organ cross-talk' among individual systems^25^. Our findings support this reciprocal interaction between the lung and other organs, as well as the contribution of extrapulmonary organ dysfunction to mortality in PARDS, which reinforces the results of previous studies^11,12,33,34^.

Increased P50, which is equivalent to a rightward shift of the hemoglobin-oxygen dissociation curve, is indicative of a decreased hemoglobin-oxygen binding affinity, which promotes oxygen unloading^35^. Factors that increase P50 include decreased pH, high levels of erythrocyte 2,3-diphosphoglycerate (2,3-DPG), and fever^36^. The complicated interplay among these various factors makes it difficult to determine the final P50. For instance, acidemia may increase P50 via the Bohr effect; however, it also reduces 2,3-DPG production, which may decrease P50. Chronic hypoxemia and anemia increase 2,3-DPG, whereas hypophosphatemia decreases 2,3-DPG.

Although this area of clinical research remains rather small, several previous studies that have investigated hemoglobin-oxygen affinity in critical illness have demonstrated conflicting results^15^. Myburgh et al. reported reduced P50 in critically ill patients^37^, whereas two recent investigations^38,39^ revealed no difference in P50 compared to the healthy population. Focusing on ARDS, an Australian group demonstrated leftward shifts of the oxyhemoglobin dissociation curves^40^. In contrast, a group of Belgium ARDS patients demonstrated a rightward shifted curve^41^, which is consistent with our findings.

In addition, our study revealed that P50 was associated with mortality regardless of initial PARDS severity, and extrapulmonary organ dysfunction largely mediated this relationship. P50 is ultimately thought to be a comprehensive reflection of increased systemic oxygen demand following increased consumption. Moreover, P50 showed a significant association with the dysfunction of several types of extrapulmonary organs. Extrapulmonary organ dysfunction was a strong risk factor for mortality in PARDS, and the mediation analysis confirmed the link between P50 and death with concurrent extrapulmonary organ dysfunction being a significant mediator independent of potential covariates, including the degree of hypoxemia. Our findings support the role of extrapulmonary organ dysfunction as a potential risk stratification system, and P50 as a likely explanation for this organ cross-talk.

The strength of this study was that it investigated P50 in association with clinical outcomes in PARDS patients. It builds upon the findings of previous studies and demonstrates causal inferences with explanatory variables, including validated criteria for organ dysfunctions. Nevertheless, we acknowledge several limitations that should be considered when interpreting the results. First, our study was conducted using a limited number of patients as this was a single-center study. Only patients who were older than 1 year and had arterial gas results of sO_2_ < 97% were included. We utilized Doyle’s method to determine P50. This method is a single-point analysis using a pair of oxygen tension and saturation measurements, which is thought to be suitable in daily practice, even for the pediatric population. Although this method had been validated using the data provided by Severinghous^42^, the calculated P50 involves fundamental limitations, as the values are estimates at best. Finally, our findings primarily focused on P50 and organ dysfunction at the time of PARDS onset, but we did not consider subsequent changes or newly developing organ dysfunctions. Further prospective validations with a larger sample size are needed to overcome the inherent limitations of this study design.

## Conclusions

In conclusion, P50, a measure of hemoglobin-oxygen affinity, may reflect the degree of extrapulmonary organ dysfunction in PARDS and may be associated with mortality. Future studies should consider P50 as a potential candidate index for risk-stratification in PARDS.

## Supplementary Information

Supplementary Information.

## Data Availability

The data that support the findings of this study are available on request from the corresponding author. The data are not publicly available because they contain information that could compromise research participant privacy.
