# Supplementary material for: P50 implies adverse clinical outcomes in pediatric acute respiratory distress syndrome by reflecting extrapulmonary organ dysfunction
Source: Sci Rep. 2022 Aug 11;12:13666. doi: 10.1038/s41598-022-18038-6 (PMC9372160; doi:10.1038/s41598-022-18038-6)
Supplement: Supplementary file 1 — Supplementary Information. [file 41598_2022_18038_MOESM1_ESM.pdf]

P50 implies adverse clinical outcomes in pediatric acute respiratory distress syndrome by reflecting extrapulmonary organ dysfunction

Yura Kim, M.D.[1], Jae Hwa Jung, M.D.[1], Ga Eun Kim, M.D.[1,2], Mireu Park, M.D.[1], Myeongjee Lee, Ph.D.[3], Soo Yeon Kim, M.D.[1]*, Min Jung Kim, M.D., Ph.D.[1], Yoon Hee Kim, M.D., Ph.D.[1], Kyung Won Kim, M.D., Ph.D.[1], and Myung Hyun Sohn, M.D., Ph.D.[1]

[1]Department of Pediatrics, Severance Hospital, Institute of Allergy, Brain Korea 21 PLUS Project for Medical Science, Yonsei University College of Medicine, Seoul, Republic of Korea

[2]Department of Pediatrics, Keimyung University School of Medicine, Keimyung University Dongsan Hospital, Daegu, Republic of Korea

[3]Biostatistics Collaboration Unit, Department of Biomedical Systems Informatics, Yonsei University College of Medicine, Seoul, Republic of Korea

Supplementary Figure 1. Patient selection flowchart.

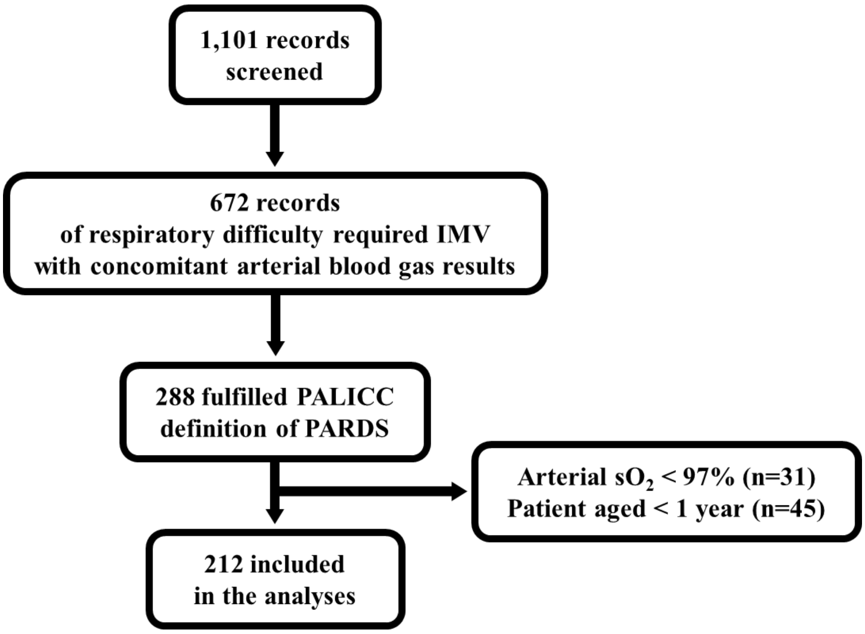

IMV, invasive mechanical ventilation; PALICC, Pediatric Acute Lung Injury Consensus Conference; PARDS, pediatric acute respiratory distress syndrome, sO2, hemoglobin oxygen saturation.

Supplementary Figure 2. Distribution of total subjects according to the number of dysfunctional extrapulmonary organs.

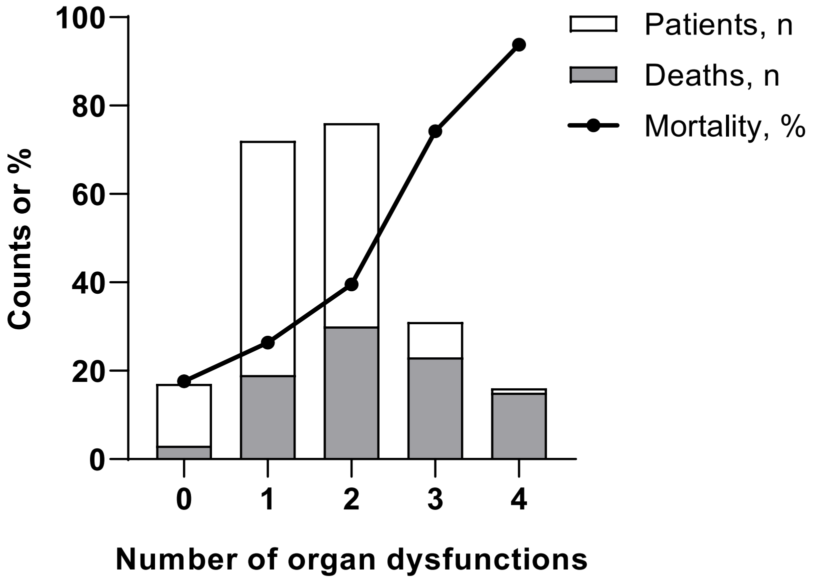

The white and gray bars represent the number of total patients and nonsurvivors, respectively. The black line denotes the percentage of mortality. Of note, mortality increases as the number of organs with dysfunction increase.

Supplementary Table 1. Multivariate regression analysis of the association between P50 and the type of organ dysfunction.

Type of organ dysfunction	Adjusted HR	95% CI	*p*-value
**Non-pulmonary**	1.186	1.129–1.245	< 0.001
**Neurological**	1.188	1.102–1.281	< 0.001
**Cardiovascular**	1.332	1.201–1.477	< 0.001
**Renal**	1.493	1.181–1.886	0.001
**Hematologic**	1.253	1.060–1.481	0.008

Analyses were adjusted for age, sex, weight, comorbidities, and initial PALICC grade.

HRs were depicted for each point change in PELOD-2 scores.

HR, hazard ratio, CI, confidence interval, PALICC: Pediatric Acute Lung Injury Consensus Conference.

Supplementary Table 2. Multivariate analysis of variables regarding the association of P50 with the type of organ dysfunction

Variable	PELOD-2 score													
	Total		Respiratory		Nonrespiratory		Neurologic		Cardiovascular		Renal		Hematologic	
	β	*P-value*	β	*P-value*	β	*P-value*	β	*P-value*	β	*P-value*	β	*P-value*	β	*P-value*
**Age, yr**	-0.022	0.828	0.033	0.042	-0.055	0.586	-0.067	0.299	0.061	0.160	-0.016	0.431	-0.033	0.258
**Sex (female vs male)**	-0.167	0.773	0.035	0.707	-0.203	0.729	-0.013	0.973	-0.050	0.842	-0.042	0.720	-0.097	0.567
**Weight, kg**	0.025	0.415	-0.005	0.299	0.031	0.329	0.014	0.474	-0.005	0.735	0.002	0.778	0.019	0.037
**Comorbidity**														
**Airway/Pulmonology**	-0.741	0.506	0.157	0.385	-0.898	0.424	-1.824	0.012	0.367	0.450	-0.178	0.435	0.736	0.025
**Oncology**	1.503	0.180	0.151	0.407	1.352	0.231	-1.561	0.031	0.492	0.314	0.175	0.444	2.246	<0.001
**Neurology**	-1.126	0.346	0.222	0.254	-1.347	0.263	-1.167	0.130	-0.293	0.573	-0.437	0.074	0.550	0.117
**Genetic syndromes**	-0.592	0.704	0.417	0.101	-1.009	0.521	-1.851	0.067	0.453	0.505	-0.350	0.272	0.738	0.107
**Hepatic failure/transplant**	0.365	0.840	0.126	0.668	0.239	0.896	-1.053	0.366	-0.052	0.947	0.299	0.418	1.045	0.050
**PARDS severity (with mild PARDS as reference group)**														
**Moderate**	-0.118	0.859	0.101	0.350	-0.219	0.743	-0.285	0.506	-0.023	0.938	0.092	0.501	-0.003	0.987
**Severe**	1.928	0.008	0.620	<0.001	1.308	0.073	0.196	0.673	0.477	0.131	0.282	0.057	0.353	0.096
**P50, mmHg**	0.182	0.001	0.024	0.007	0.158	0.004	0.066	0.056	0.075	0.001	0.022	0.046	-0.005	0.742

PELOD-2: Pediatric logistic organ dysfunction-2, PARDS: Pediatric acute respiratory distress syndrome
